# Study on the Health Status and Health Service Utilization of the Elderly of a Remote and Poor Village in a Mountainous Area in Jinzhai, Anhui

**DOI:** 10.3390/ijerph14040408

**Published:** 2017-04-12

**Authors:** Chaoqun Hu, Wenya Yu, Yipeng Lv, Haiping Chen, Qiangyu Deng, Lulu Zhang

**Affiliations:** Institute of Health Management, Second Military Medical University, Shanghai 200433, China; hu_jayz@outlook.com (C.H.); jsjyyuwenya@sina.cn (W.Y.); epengl@163.com (Y.L.); chenhaipingyx@163.com (H.C.); smmudqy@163.com (Q.D.)

**Keywords:** the elderly, health service utilization, health status, mountainous areas, poor people

## Abstract

*Background*: Despite the rapid development of China’s economy, a number of poor areas in China continue to exist. The health status of the elderly in the poor areas is a matter of concern. This study aims to explore the status of the elderly in terms of their health status, health service needs, and utilization among rural residents of a remote and poor village in a mountainous area in Jinzhai, Anhui. Furthermore, this study aims to explore the differences between the nation rural area average level and the remote and poor village in the mountainous area in terms of health status and health service utilization. *Methods*: Cluster sampling was used to obtain the sample. A total of 110 elderly people were selected from the village, and face-to-face interviews were conducted with questionnaires by trained investigators to collect data. *Results*: All items except vision, language disability, and self-care disability were found to be higher than the national average level. In terms of mental health, Zishu Village has a ratio of 44.1% for the symptoms of anxiety and depression, which is higher than the average for the national rural areas. The two-week prevalence rate, prevalence of chronic diseases, and non-hospitalization rate of those who need hospitalization (%) in Zishu Village was 62.7%, 88.2%, and 47.6% respectively, which was higher than the rural values of the National Survey (2008). Most of the outpatient visits were to the village clinics, while the hospitalizations were mainly to county hospitals. The two-week visiting rate was 24.1%, which was lower than 2008. The hospitalization rate in Zishu Village was 10.8%, which is similar to the level of 2008. *Conclusions*: The health level and the utilization of health services of the people in Zishu Village, Jinzhai, are generally lower than the national average. Financial difficulties continue to remain the major factor affecting the utilization of hospitalization services of this remote and poor village in Jinzhai, Anhui.

## 1. Background

Economy is an important factor that affects human health. In the past, China’s economic development has been greatly successful. However, China continues to be a developing agricultural country, whose economic development faces some relative imbalance with the coastal economy being significantly better than the inland economy with some extent of economic backwardness in rural poor areas. China implemented the Precision Poverty Alleviation Program in 2015, hoping to alleviate poverty in all parts of the country. The latest data by the State Council reveals the existence of 592 poor counties, 128 thousand poor villages, and approximately 13.5 million elderly people in a total population of 70 million poor people (by the end of 2016, China’s total population was 1.38271 billion people, with the number of people aged 60 and above being 230,860,000, accounting for 16.7% of the total population). Comparing with the World Bank’s latest statistics, in 2014, China’s healthcare spending at 5.7% of GDP was far from 17.1% in the United States. In China, governments have paid less attention to health service development in rural areas than that in urban areas, and the amount of funding available for primary health care in economically depressed rural areas is less than that in urban areas and coastal regions, which has resulted in a relatively weak rural health system [1,2,3]. In 2015, infant and maternal mortality were much higher in rural than in urban areas (9.6 vs. 4.7 and 20.2 vs. 19.8 per 100,000, respectively). We can now identify a widening gap in access to health care with the economy developing rapidly over the past 30 years [4].

Jinzhai, one of the 592 counties identified as poor, is the largest and most populous county in Anhui, China, with 680,000 people. It is an economically backward agricultural area in Western China. Jinzhai is one of the most poverty-stricken counties and among the key counties identified in the Dabie Mountain Area Poverty Alleviation in 2011. At the end of 2015, the situation was as follows: there were still 84,300 poor people (approximately 16,000 elderly people), poverty alleviation and development tasks were arduous, and development was a very difficult task. In 2014, the average net income of all residents in Jinzhai was 7762 RMB (approximately US$1108), but the national level of wage and per capita consumption expenditure were approximately 20,167 RMB (US$2881) and 14,491 RMB (US$2070), respectively. We can speculate that the income of the poor was lower and that the conditions of their life became worse. According to the Poverty Monitoring Report on Rural China 2010, the percentage of Chinese peasants who were unable to receive appropriate health services owing to financial reasons only dropped by 7.8% from 2002 to 2009 [5]. Lower economic status can worsen nutrition, housing, and recreational opportunities. It could be inferred that the poor people in Jinzhai, particularly in a remote and poor countryside in a mountainous area, face a greater number of public health problems compared to other areas in China. However, the New Cooperative Medical System (NCMS), a new type of rural health insurance supported by the government, provides overall coverage, which is a primary medical security system for rural China to improve access to health services and prevent impoverishment due to medical expenses. In this system, personal contributions to the insurance premium are relatively low, with allowances from the local and central government. In 2016, this medical security system covered more than 97% of the rural residents. The scope of the policy of the outpatient and inpatient reimbursement ratio was 50% and 75%, respectively, which was not adequate to solve their medical problems [6]. In those areas, low income, poor health consciousness, and traffic inconvenience remain significant barriers to health service utilization for the local populace.

Prior studies have not only explored the health status and health service utilization in rural areas, but also explored the impact of predisposing, enabling, and need factors in the utilization of health services among rural residents [7,8,9,10,11]. Studies from the USA [12] and Poland [13] suggest disparities in health status and health service use between rural and urban areas. Similar findings have been observed in Africa; for instance, in Zambia, people in urban areas used health services more than people in poor rural areas [14]. Researchers from Africa also found that factors such as distance to health facilities and the level of education are key in affecting healthcare utilization [7,9,15]. However, few studies have focused on people in remote and poor areas, particularly elderly people in mountainous areas. Our study aims to report the self-reported health status and service utilization of the elderly people in a remote and poor village in a mountainous area in Jinzhai, Anhui.

## 2. Methods

### 2.1. Sampling

The study was carried out in 2016 in Zishu Village, a key poor village in Jinzhai, which is located in the hinterland of the Dabie Mountains, on the border of Anhui, Hubei, and Henan provinces. Cluster sampling was used to obtain the sample. The investigation was conducted on the basis of cooperation with the local government. Respondents were excluded from the current study if they met at least one of the exclusion criteria: (1) aged 60 years or above; (2) had lived in Jinzhai for more than six months; (3) adequately aware enough (in terms of mental stability) to answer the questionnaire. Prior to conducting the investigation, a village cadre helped us communicate with the eligible respondents, and a written consent form was obtained from all the participants before the interview. It was clarified that participating in the study was entirely voluntary. After exclusion based on eligibility criteria, a total of 149 elderly people were found to be eligible, and 110 expressed their willingness to participate in our survey. Out of the remaining 39, a total of 12 elderly people could not be contacted, 11 had no time, and 16 were not willing to participate because of the privacy. Five postgraduate students from Science of Social Medicine and Health Management at the Second Military Medical University participated in a three-day training course, which mainly included imparting skills to communicate with the local community and to operate and record scale questions (such as regarding how to judge vision, hearing, etc.) prior to the investigation. They were trained uniformly by a survey supervisor from the Second Military Medical University so that they would be able to administer the questionnaires competently and ensure consistent quality. The village cadre was responsible for guiding and organizing the local people to participate, and for recording the participants’ information to avoid repetition in the survey.

### 2.2. Questionnaire

Our survey was used in the Fourth National Health Service Survey (2008 edition) which primarily covers the following aspects: (1) personal information and physical status of informants, including the socioeconomic characteristics, self-assessment of the health status, disease prevalence, disabilities, and health risk factors; (2) health service needs and utilization, including treatment for any disease, degree of satisfaction of demands, reasons for dissatisfaction; utilization of the public health services, outpatient and emergency departments, and hospitalization; and the payment of medical expenditures; and (3) the degree of satisfaction of the villagers with the service system, service-providing processes, and the coverage and level of medical insurance. The item “illness within two weeks” in the present survey was defined as the self-assessment of the presence of a disease based on the healthcare service aspect, and the participants with at least one of the following characteristics were considered to be suffering from “illness within two weeks”: (1) perception of physical discomfort in the two weeks prior to the survey, consultation with medical and health units, and confirmation of disease and treatment; (2) experience of feeling uncomfortable in the two weeks prior to the survey, not approaching medical treatment units, but taking self-medication or some form of supplementary treatment; (3) experience of feeling uncomfortable in the two weeks prior to the survey but not consulting medical settings or self-prescribing any form of treatment, and stopping work or staying in bed for at least one day. The item “two-week prevalence” was estimated with “illness within two weeks” item and was described as the percentage of the participants with illness in the two weeks prior to the survey. The item “two-week visiting rate” was estimated based on the frequency of the participants obtaining a healthcare facility for healthcare services in the two weeks prior to the survey due to discomfort and was described as the person-time of seeking medical care within the past two weeks. The “hospitalization rate” was estimated based on the hospitalization of the subjects within the past year and was described as the person-time of hospitalization in the past one year due to illness. Cronbach’s alpha for the overall questionnaire was 0.719. The Kaiser-Meyer-Olkin value was 0.702, and the results of Bartlett’s test were significant (normal approximate = 1179.958, *p* < 0.001).

### 2.3. Data Collection

Face-to-face interviews were conducted by staff at the participants’ homes. Each staff member was trained by professional investigators from the university. Quality control and guidance personnel were present during the interview process, and a trained director conducted further validity checks in order to guarantee the accuracy of the final completed questionnaires.

Hearing was judged based on whether respondents could hear questions clearly in the process of investigation, and participants responded by selecting one of the following: “Difficult to hear clearly”, “Can hear clearly when you raise your voice” and “Can hear clearly.” Sight was judged based on the difficulty level expressed for identifying an acquaintance at a distance of 20 m (respondents can answer the case when wearing glasses). Participants responded by selecting one of the following: “Difficult to see clearly”, “A little fuzzy but can be identified” and “Can see clearly”. The question used to examine language ability was “Have you experienced any difficulty in speaking in the last six months?” and responses were collected in the form of “Yes” or “No”. The question used to examine mobility ability was, “What’s your current state of mobility ability?” and participants responded by selecting one of the following: “Can walk normally” and “Difficult in walk normally (such as walking with crutch) or being bedridden”. The question used to examine self-care ability was, “Can you take care of yourself (such as dressing, bathing, and going to the toilet)?” and responses were collected in the form of “Yes” or “No”. The question used to examine the ability to perform daily activities was, “Can you perform daily activities (such as work, read and do housework) normally?” and responses were collected in the form of “Yes” or “No”. The question used to examine pain/discomfort, anxiety/depression was, “Do you feel pain/discomfort?” or “Do you feel anxiety/depression?” and responses were collected in the form of “Yes” or “No”.

### 2.4. Data Process and Statistical Analyses

This study compares the results of the National Health Survey for rural areas with the survey conducted in Zishu Village on different aspects. Two investigators were asked to input the data with EpiData3.1 software (EpiData Association, Odense, Denmark) independently. The data were checked and then output into an EXCEL database, and analyzed with SAS 9.4 software (SAS Institute Inc., Cary, NC, USA). A chi-square test was used for the analyses. *p* ≤ 0.05 was considered to be statistically significant.

### 2.5. Ethical Statement

All the participants provided their informed consent for inclusion prior to participating the study. The study was conducted in accordance with the Declaration of Helsinki, and the protocol was approved by the Ethics Committee of Second Military Medical School and the ethical approval code was 2013LL058.

## 3. Results

The questionnaire survey was administered in Mandarin to 110 potentially eligible individuals, out of whom three were unable to communicate in Mandarin. The remaining 107 participants completed the questionnaires, five of which were incomplete. Thus, 102 valid subjects were analyzed in this study. The valid response rate was 92.7% (102/110).

Descriptive analysis by socio-economic status is presented in Table 1. The per capita income of the sample population was 4834 RMB in 2016, which is similar to the national rural average in 2008 (4932 RMB). More than half (58.8%) were female, and 69.6% were married. The age range was as follows: (60–69)—54.9%; (70–79)—39.2%; above 80—5.9%.

Descriptive analysis by health status is shown in Table 2. Health condition can be primarily divided into physical and mental health. The findings of the present survey showed that respondents with difficulty in listening, vision, personal language, mobility ability, self-care ability, daily activity ability, and those who experience pain and discomfort was respectively 38.8%, 43.8%, 24.5%, 43.2%, 13.8%, and 33.3%. Above all, all items except vision, language disability, and self-care disability were found to be higher than the national average level. In terms of mental health, Zishu Village has a ratio of 44.1% for the symptoms of anxiety and depression, which is higher than the average level of the national rural areas.

Table 3 shows a comparison of the health service needs and utilization in the national survey (2008) and Jinzhai survey. The two-week prevalence rate, prevalence of chronic diseases, and non-hospitalization rate for those who needed hospitalization (%) in Zishu Village was 62.7%, 88.2%, and 47.6%, respectively, which was higher than the National Survey (2008) values. The two-week visiting rate was 24.1%, which was lower than 2008. The hospitalization rate in Zishu Village was 10.8%, which is similar to the level of 2008 (Table 3).

Table 4 shows Village clinics were the major choice for the villagers in two surveys, and the percentage in Zishu Village was 71.8%, which was higher than the value of 2008. However, the percentages of township hospitals and county hospitals were found to be 12.8% and 2.6% in this study, respectively, which were decreased compared with the level of 2008. The percentage of city hospitals or above was higher than the level of 2008.

## 4. Discussion

Our study shows that in terms of language, hearing, and vision disability rate, the disability rate of the elderly population in Zishu Village, Jinzhai, is higher than that in the rural areas of China in three aspects. We found that elderly residents in Zishu Village had higher levels of mobility disability and daily activity disability, and experienced a higher level of pain and discomfort excepting when taking care of themselves, compared with the national rural level. Additionally, there is no statistical difference in taking care of oneself between the average level of Zishu Village and the country’s rural areas. We can draw a conclusion that the overall health of the elderly in Zishu Village is worse than that of the country. The reasons for the significantly worse health state may be the result of geographical position, economic condition, and educational level. Firstly, Zishu Village is located in a remote mountainous area, where transportation is not convenient and medical health resources are far. Prior research shows that physical distance from healthcare facilities is an important determinant of health [7,15,16]. Secondly, personal or familial economic difficulty is a major problem, which may lead to poor living habits and difficulty in seeking health services. Thirdly, education level must be considered. The illiteracy rate of respondents in Zishu Village is approximately 63.7%, but the Sixth Census reveals that the nation level of illiteracy rate is 26.3%. The overall educational level of the elderly in Zishu Village is lower than the average level of the whole country. Lower education may lead to a lack of knowledge of preventive health care and awareness of health services, which may result in a poorer health state [17,18]. Additionally, an unhealthy mental state may occur due to the departure of their children, which makes them lack emotional support and psychological comfort and makes them vulnerable to falling into a state of boredom, loneliness, and helplessness [19]. Furthermore, loneliness itself has been confirmed to be associated with an increased risk of mortality and cardiovascular disease [20,21,22], elevated blood pressure and cortisol [23,24], heightened inflammatory responses to stress [25,26], and falling into depression and fatigue [27], which forms a vicious circle. Therefore, the elderly residents in Zishu Village are in a poor health state. In our study, we found a unique phenomenon in terms of the rate of disability of taking care. The rate of disability of taking care of poorer physical functioning status of the elderly in Zishu Village was of no statistical significance in the national rural areas, which may be caused by the phenomenon of rural-to-urban migration of workers in China. In China, large numbers of young people in economically backward rural areas immigrate to developed regions for work, and are called “rural-to-urban migrant workers”. Rural-to-urban migrant workers have given rise to a phenomenon where no one takes care of the elderly or of children in rural areas of China. This may compel a greater number of elderly people in poor areas in the same physical condition as those in developed areas to take care of themselves. Therefore, the elderly can only take care of themselves in order to undertake more daily work to ensure their bodies get exercise regularly. That may be the reason why the ability of the elderly to take care of themselves is not lower than the general level of the country [28].

The two-week prevalence of the elderly in Zishu was 62.7% according to the present survey, which is much higher than the national level of the people in the village in 2008 (37.8%). The two-week visiting rate also revealed a significant gap, too. The value of the present survey was 37.3%, which was significantly lower than the national rural level (62.2%). Compared with the national rural level (60.5%) in 2008, our present survey showed 99% coverage of NCMS. Those findings were different from the findings of the current study, which reveal that the implementation of NCMS increased the two-week visiting rate [29]. Contrarily, the hospitalization rate was 10.8% in the present survey, which was very close to the national level in 2008 (12%). This is consistent with the findings of a study by Han et al. [30]. Thus, NCMS has improved the utilization of hospitalization services but not outpatient services by villagers; in addition, the non-hospitalization rate for those who needed hospitalization in Zishu Village (47.6%) is much higher than the national level in 2008 (26.3%), implying financial difficulties continued to remain the major factor affecting the utilization of hospitalization services of villagers.

The results of the two surveys showed that the utilization of health services of two-week visits was dominant in village clinics, and the percentage was even higher than the average national rural level, which could be because the coverage of NCMS in village clinics is significantly higher than that in others. Furthermore, on the one hand, Jinzhai is a remote mountainous rural area where traffic is not convenient; on the other hand, the development of village clinics has achieved great success, which is very convenient for the villagers, but a greater number of villagers have sought health services from village clinics.

The main limitation of our study is that a number of subjective indicators were adopted in the questionnaire. We did not use the professional scale to measure the physical and psychological state of the participants, which might have caused a bias in the findings. Secondly, limited by terrain and human resource constraints, the small study sample size might have precluded finding significant associations among variables. Further research with normal controls and larger sample sizes are required. Thirdly, although we added some international studies in the discussion section, the overall discussion is based on Chinese data and studies because of the characteristics of rural China. Finally, because the national health service survey data is not updated, we used the survey data of 2008 to make a comparison. Nevertheless, the findings of our study have important implications. On the one hand, these findings may be useful for promoting the development of a national health policy for the elderly in remote and poor villages, particularly in mountainous areas, a growing public health concern in our aging society. On the other hand, our study provides a warning to rural-to-urban migrant workers that it is necessary to pay close attention to the elderly and to visit family to the greatest extent possible.

## 5. Conclusions

The health status and the utilization of healthcare services in Zishu Village, Jinzhai, are lower than the national average. Although the NCMS policies have alleviated the medical burdens of villagers to a certain extent, financial difficulties continue to remain a major factor affecting the utilization of hospitalization services of villagers. Because of the popularity of village clinics, the utilization of health services of two-week visiting was dominant in village clinics in Zishu Village, and the percentage was even higher than the average national rural level.

## Figures and Tables

**Table 1:** Comparison between the two surveys: National and Jinzhai.

Subjects	2008 (National Survey)	2016 (Jinzhai Survey)	χ^2^	*p*-Value
N	29,634	102	-	-
Revenue	4932	4834	-	-
Gender				
Male	13,839 (46.7%)	42 (41.2%)	1.256	0.264
Female	15,795 (53.3%)	60 (58.8%)		
Age (in year)				
60–69	16,921 (57.1%)	56 (54.9%)	4.215	0.122
70–79	9453 (31.9%)	40 (39.2%)		
>80	3260 (11%)	6 (5.9%)		
Marital status				
Single	711 (2.4%)	3 (2.9%)	0.99	0.804
Married	20,033 (67.6%)	71 (69.6%)		
Separated	207 (0.7%)	0 (0%)		
Widowed	8623 (29.1%)	28 (27.5%)		
NCMS	17,929 (60.5%)	101 (99%)	61.58	<0.001

NCMS: New Cooperative Medical System.

**Table 2:** Comparison between the two surveys in terms of health status: National and Jinzhai.

Subjects	2008 (National Survey)	2016 (Jinzhai Survey)	χ^2^	*p*-Value
Physical Status				
Hearing				
Hard ^1^	2311 (7.8%)	15 (14.3%)	7.084	0.029
Slight ^2^	7053 (23.8%)	25 (24.5%)		
Sight				
Extreme ^3^	1393 (4.7%)	13 (12.2%)	16.413	<0.001
Moderate ^4^	8238 (27.8%)	32 (31.6%)		
Language disability	4445 (15.0%)	25 (24.5%)	7.198	0.007
Mobility disability ^5^	4949 (16.7%)	44 (43.2%)	50.849	<0.001
Self-care disability	3438 (11.6%)	14 (13.8%)	0.447	0.504
Daily activities disability	4860 (16.4%)	34 (33.3%)	21.198	<0.001
Pain/discomfort	7112 (24.0%)	77 (75.4%)	147.019	<0.001
Mental health				
Anxiety/depression	4682 (15.8%)	45 (44.1%)	60.971	<0.001

^1^ Difficult to listen clearly even if someone raises their voice. ^2^ Listening clearly by raising voice. ^3^ Could not identify an acquaintance who was 20 m away. ^4^ Could identify an acquaintance 20 m outside but could not see clearly. ^5^ Difficulty in walking normally or being bedridden.

**Table 3:** Comparisons of the health service needs between the two surveys.

Subjects	National Survey	Jinzhai Survey	χ^2^	*p*-Value
Two-week prevalence (%)	11,202 (37.8%)	64 (62.7%)	26.88	<0.001
Two-week visiting rate (%)	5825 (52.0%)	15 (24.1%)	20.79	<0.001
Prevalence of chronic diseases (%)	11,528 (38.9%)	90 (88.2%)	103.92	<0.001
Hospitalization rate (%)	3556 (12%)	11 (10.8%)	0.14	0.7061
Non-hospitalization rate for those who needed hospitalization (%)	1269 (26.3%)	49 (47.6%)	24.09	<0.001

**Table 4:** Percentages of visits in the hospitals at different levels (%).

Subjects	National Survey	Jinzhai Survey
Village clinics	6419 (57.3%)	46 (71.8%)
Township hospitals	2733 (24.4%)	8 (12.8%)
County hospitals	1714 (15.3%)	2 (2.6%)
City hospitals	146 (1.3%)	3 (5.1%)
Provincial hospitals	78 (0.7%)	2 (2.6%)
Others	101 (0.9%)	3 (5.1%)
**χ^2^**	32.90	
*p*	<0.001	
